# Trade‐Offs in the Moonlight: Influence of Interspecific Pressures, Temperature, and Moon Phases on the Activity Patterns of Asiatic Brush‐Tailed Porcupine

**DOI:** 10.1002/ece3.73303

**Published:** 2026-03-19

**Authors:** Haidong Zhou, Wenbo Yan, Zhigao Zeng, Shaoliang Xue, Qi Wang, Chunshen Liang, Lu Liang

**Affiliations:** ^1^ School of Biological Science and Engineering Shaanxi University of Technology Hanzhong China; ^2^ State Key Laboratory of Animal Biodiversity Conservation and Integrated Pest Management, Institute of Zoology Chinese Academy of Sciences Beijing China; ^3^ Jianfengling Branch of the Management Office of Hainan Tropical Rainforest National Park Ledong China

**Keywords:** activity rhythms, *Atherurus macrourus*, interspecific pressure, lunar cycle

## Abstract

Understanding the chronobiological adaptations of wildlife to diverse environmental stressors is crucial for effective conservation. This study described the activity patterns of the Asiatic brush‐tailed porcupine ( *Atherurus macrourus* ) in the Jianfengling area of Hainan Tropical Rainforest National Park, using extensive infrared camera trap data collected from 2020 to 2022. We analyzed the combined effects of lunar cycles, temperature gradients, and interspecific interactions—including competition with the ferret‐badger ( *Melogale moschata* ), predation by the leopard cat ( *Prionailurus bengalensis* ) and disturbance from domestic dogs ( *Canis lupus familiaris* ). Kernel Density Estimation (KDE) for activity pattern characterization, Relative Activity Index (RAI) for intensity quantification, and negative binomial Generalized Linear Models (GLMs) to disentangle multifactor synergies are used. Results showed a primarily nocturnal pattern with peak activity between 20:00 and 22:00, along with strong avoidance of full‐moon phases and a preferred temperature range of 15°C–22°C. Temporal segregation may reduce direct resource competition, and behavioral flexibility was observed in response to varying predation risks and human disturbances. These adaptive strategies represent key trade‐offs among foraging efficiency, predator avoidance, and thermoregulation, demonstrating the species' behavioral plasticity. Thus, this study provides essential empirical evidence for developing conservation strategies to protect endangered species in the face of increasing climate change and human encroachment in tropical regions.

## Graphical abstract

Using camera trap data from a tropical rainforest, we show that the Asiatic brush‐tailed porcupine uses behavioral trade‐offs: avoiding full moons, preferring 15°C–22°C, and shifting activity to reduce conflict with predators/competitors. This plasticity reveals how it balances foraging efficiency and survival risks, offering insights into conserving wildlife under synergistic environmental pressures.

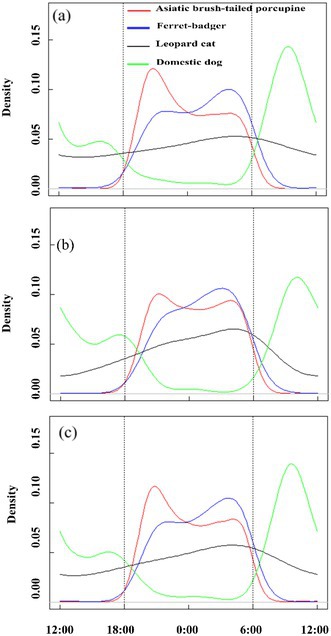

## 1 Introduction

Activity patterns, representing phylogenetically conserved behavioral adaptations synchronized with environmental periodicity, constitute fundamental biological mechanisms for fitness maximization (Halle and Stenseth 2000). This biological framework provides critical insights into animal‐environment interplay, particularly regarding ecological niche optimization and utilization strategies. The emergence of such temporal architectures originates from the dynamic integration of evolutionary heritage and environmental entrainment (Zhang 1995; Shi et al. 2011), governed through multilayered regulatory systems encompassing endogenous determinants (ontogenetic progression, sexual differentiation) and exogenous parameters (predatory threats, competitive exclusion, anthropogenic disturbances), achieving precise synchronization with photoperiodic and seasonal cycles (Shang 2006; Duan 2014). Empirical investigations reveal that rodent species exhibit pronounced thermo‐photic sensitivity, dynamically fine‐tuning their activity patterns and foraging strategies in response to fluctuating environmental parameters such as photic intensity and thermal gradients (Gliwicz and Dąbrowski 2008). The lunar cycle paradigm demonstrates this environmental synchronization, revealing taxon‐specific behavioral adaptations: visual predators exhibit lunarphilic optimization, amplifying nocturnal activity and hunting efficacy during heightened lunar illumination (Prugh and Golden 2014; Tang et al. 2017; Jiang et al. 2023), while small prey exhibit lunar‐phobic avoidance strategies, prioritizing nocturnal activity during lunar occultation (low moonlight) to mitigate predation vulnerability as reduced visibility minimizes detection by visual predators, with brief crepuscular supplementary activity only under high predation risk (e.g., full moon) while small prey exhibit lunar‐phobic avoidance strategies: their primary activity is nocturnal, prioritized during lunar occultation (low moonlight) to reduce detectability by visual predators; brief crepuscular supplementary activity occurs exclusively under high predation risk (e.g., full moon)—a compensatory strategy when nocturnal predation risk is elevated (Bowers 1990; Prugh and Golden 2014; Shi et al. 2021).

Lunar cycles—defined as the periodic changes in moonlight intensity resulting from the relative positions of the Earth, Moon, and Sun—serve as a critical abiotic driver regulating wildlife behavior (Prugh and Golden 2014). Typically categorized into five consecutive phases (new moon, waxing crescent, full moon, waning gibbous, waning crescent), these phases are further grouped by illumination intensity into low (new moon + waning crescent), medium (waxing crescent + waning gibbous), and high (full moon) levels (Sutherland and Predavec 1999). This moonlight gradient directly modulates the foraging efficiency of visual predators and the predation risk of prey: brighter moonlight enhances predators' ability to detect prey, while dimmer conditions provide prey with concealment (Jiang et al. 2023; Tang et al. 2017). For nocturnal species like the Asiatic brush‐tailed porcupine, this gradient creates context‐dependent selective pressures, shaping how they balance foraging opportunities against predation risk across lunar conditions. Despite this well‐documented ecological link, few studies have integrated lunar phase effects with other biotic (predation, competition) and abiotic (temperature) factors to explore their combined influence on tropical mammals' adaptive strategies—this gap further justifies the present research.

The Asiatic brush‐tailed porcupine ( *Atherurus macrourus* ) emerges as the predominant Hystricidae species within the Jianfengling region of Hainan Tropical Rainforest National Park (Li et al. 2002). Functioning as keystone primary consumers in tropical biomes, these porcupines orchestrate vegetative resource regulation through selective herbivory while facilitating zoochoric propagation via seed dispersal. Their excavated subterranean refugia further serve as critical microhabitat subsidies for commensal fauna, collectively elevating regional biodiversity through niche construction (Xie et al. 2009; Kitamura and Poonswad 2010). Exhibiting heightened sensitivity to thermal and hydrological parameters, these rodents' demographic fluctuations and ethological plasticity render them potent bioindicators for ecosystem health diagnostics (Gliwicz and Dąbrowski 2008). Anthropogenic climatic forcing, manifesting through escalating nocturnal thermal regimes, now mandates critical behavioral adaptations in porcupines, including temporal compression of activity windows and chronobiological displacement of foraging activities, thereby exacerbating their ecological precarity through desynchronization from evolved thermoregulatory strategies and resource phenology.

The ferret‐badger ( *Melogale moschata* ) exhibits remarkable morphological convergence and trophic niche overlap with the Asiatic brush‐tailed porcupine. Within the Jianfengling region of Hainan Tropical Rainforest National Park, this mustelid demonstrates extensive spatial congruence with porcupine populations across altitudinal gradients, emerging as a formidable competitor for trophic resources and territorial occupancy (Zheng and Song 2010). Based on long‐term infrared camera trap monitoring data in the study area, other potential competitor species either show extremely low activity rhythm overlap with the porcupine or have excessively small population sizes—rendering their competitive effects negligible. Thus, the ferret‐badger was ultimately selected as the core competitor species for inclusion in the analysis. Regarding predation pressure, the leopard cat ( *Prionailurus bengalensis* ), a felid apex mesopredator within this ecosystem, is the sole species observed to have a definite predation relationship with the Asiatic brush‐tailed porcupine, as local carnivore taxa are scarce. This species imposes significant mortality pressure on porcupine populations through specialized hunting strategies. More critically, free‐ranging domestic canines ( *Canis lupus familiaris* ), functioning as anthropogenic vectors of ecological disruption, perpetrate multifaceted ecological perturbations including direct predation, pathogen transmission, and chemical communication interference across wildlife communities—impacts that are particularly acute for porcupine populations (Vanak and Gompper 2009). These synergistic pressures (competitive exclusion from ferret‐badgers, predation pressure from leopard cats, and anthropogenic disturbance from domestic dogs) may drive adaptive shifts in the survival strategies and activity patterns of the Asiatic brush‐tailed porcupine in this tropical rainforest ecosystem.

The nocturnal habits and secretive behaviors of the Asiatic brush‐tailed porcupine, ferret‐badger, and leopard cat, combined with their occupation of ecologically intricate nocturnal niches, present formidable challenges to direct observational studies, resulting in a paucity of data on sympatric species interactions and environmental drivers of chronobiological trade‐offs. We selected infrared camera traps specifically because (1) it avoids disturbing the shy porcupine, capturing natural activity; (2) its 24/7 continuous operation over 2 years tracks dynamic factors (lunar cycles, temperature) and rare interspecific interactions; (3) it adapts to the rainforest's hyperthermal/humid conditions, covering diverse habitats. While widely used, it uniquely addresses our study's specific challenges (Nichols et al. 2011). Tropical rainforests, constituting core components of global biodiversity hotspots, present a singular observational platform for studying animal activity rhythms through their hyperthermal and hyperhumid environments intertwined with complex interspecific interaction matrices (Malhi et al. 2014). However, current studies on animal activity rhythms in tropical rainforests have addressed the roles of individual factors such as moonlight or temperature (Wen et al. 2016; Shi et al. 2021), but lack integrated analyses of multifactor interactions. (Halle and Stenseth 2000). This limitation in research paradigms prevents a comprehensive understanding of species' adaptive strategies when facing multidimensional pressures in complex ecosystems—this constitutes the core research gap that the present study aims to address.

Consequently, this investigation employed camera trapping methodology within the Jianfengling region of Hainan Tropical Rainforest National Park to examine the synergistic orchestration of polyadic environmental determinants—lunar periodicity, thermal gradients, anthropogenic disturbances, predation pressure, and competitive interactions—upon the chronobiological adaptations of the Asiatic brush‐tailed porcupine. We aimed to answer the following research questions:

1. Predation risk‐foraging efficiency trade‐off: Under coexisting pressure of predation risk and human disturbances, how do Asiatic brush‐tailed porcupines balance lunar exposure risks against foraging benefits? Is their suppressed activity during full‐moon periods counterbalanced by crepuscular activity to maintain energetic equilibrium?
2. Interspecific competition‐resource acquisition trade‐off: Does temporal partitioning from their competitor, the ferret‐badger, result in reduced efficiency of resource acquisition?
3. Thermal adaptation‐behavioral strategy trade‐off: What physiological costs and adaptive limitations might the behavioral thermoregulatory strategies of brush‐tailed porcupines face under prolonged environmental change?

## 2 Study Area and Methods

### 2.1 Study Area

Located in the southwestern part of Hainan Island, China (108°46′–109°45′ E, 18°23′–18°50′ N), the Jianfengling area of Hainan Tropical Rainforest National Park covers a total area of 466.67 km^2^ and is administered by the park's Jianfengling Branch. The region experiences a typical tropical monsoon climate, characterized by distinct wet and dry seasons. The mean annual precipitation is 2651 mm, with the majority of rainfall occurring between May and October, while the dry season extends from November to April. The area exhibits concurrent rain and heat patterns, with an average annual temperature of 24.5°C. The average temperature of the coldest month is 19.4°C, and that of the warmest month is 27.3°C. Food availability varies seasonally: wet season has abundant fruits/new leaves, dry season has reduced fruits but persistent bark, evergreen leaves, and tubers (Li et al. 2020), ensuring no seasonal scarcity. Official records (Hainan Meteorological Bureau 2022) indicate a mean annual relative humidity of 83% ± 4% for this station, consistent with the hyperhumid characteristics of tropical rainforest ecosystems in the region. Ongoing conservation initiatives in the park include: (1) native vegetation restoration and canopy protection to maintain stable microclimates; (2) a long‐term infrared camera monitoring network for wildlife population tracking; and (3) free‐ranging domestic animal control and core habitat disturbance restriction. As a key component of the latter anthropogenic disturbance, the free‐ranging domestic dogs in the study area are primarily semi‐domesticated animals owned by local residents (living in villages adjacent to the park boundary)—not feral strays or pack‐living wild dogs. These dogs are not confined to households and roam freely within and around the park day and night, maintaining associations with human settlements while frequently accessing core wildlife habitats, thus serving as an effective proxy for human activities such as residential encroachment and occasional resource use within the park. The terrain is predominantly mountainous, with elevations ranging from 112 m to 1412 m. Dominant vegetation types include tropical evergreen monsoon forest, tropical montane rainforest, tropical semi‐deciduous monsoon forest, and tropical valley rainforest. Key plant species in the park include Longan ( *Dimocarpus longan* ), star fruit ( *Averrhoa carambola* ), elephant ear fig tree (*F. auriculata*), guava ( *Psidium guajava* ), *Hainan hydnocarpus* (*Hydnocarpus hainanensis*), which provide critical food resources (fruits, bark, leaves) and shelter for the Asiatic brush‐tailed porcupine (Mo et al. 2019, 2021). The Jianfengling area of Hainan Tropical Rainforest National Park hosts abundant animal resources and serves as a habitat for many rare species, including 215 species of birds, 68 species of mammals, 50 species of reptiles, and 38 species of amphibians. Among them, notable rare animals include: Hainan Peacock‐Pheasant (*Polyplectron katsumatae*), Hainan Partridge ( *Arborophila ardens* ), Chinese Pangolin ( *Manis pentadactyla* ), Asian Water Monitor ( *Varanus salvator* ), White‐eared Night Heron ( *Gorsachius magnificus* ), Leopard Cat ( *Prionailurus bengalensis* ), Common Palm Civet ( *Paradoxurus hermaphroditus* ), Black Giant Squirrel ( *Ratufa bicolor* ).

### 2.2 Camera Trapping

**FIGURE 1:**
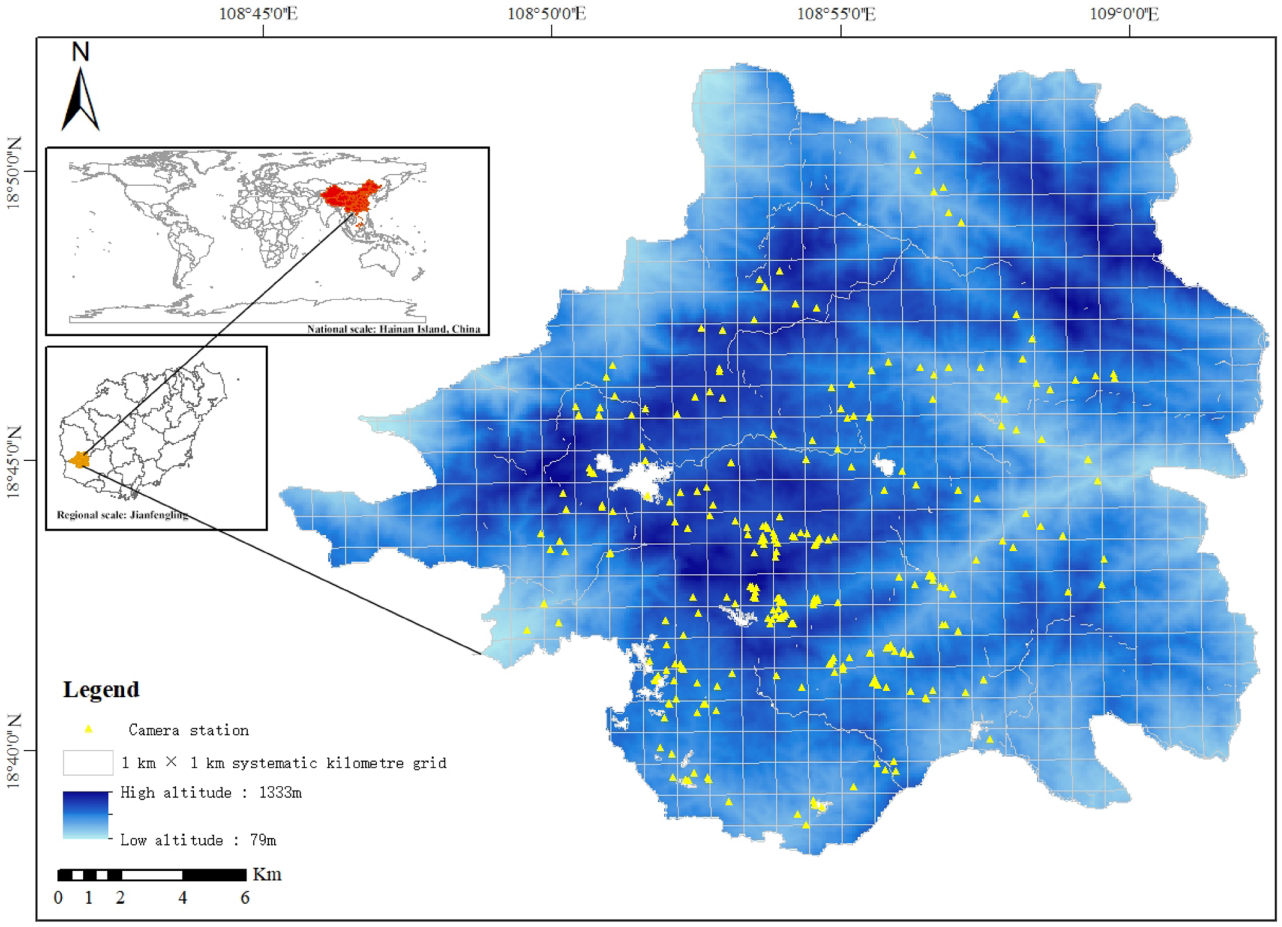
Distribution of infrared camera traps in the Jianfengling area of Hainan Tropical Rainforest National Park from 2020 to 2022. The blue‐based color‐blind‐friendly gradient represents the elevation of sampling sites (unit: Meters above sea level, m a.s.l.): Light blue (≤ 400 m a.s.l.), medium blue (400–800 m a.s.l.), and dark blue (≥ 800 m a.s.l.). Camera trap symbols are yellow triangles with 70% transparency to avoid clustering. Grid lines are light gray with 50% transparency to prevent obscuring spatial patterns. The distribution follows a 1 km × 1 km systematic grid‐based design, ensuring coverage of different elevation zones and major vegetation types.

In the Jianfengling forest area, we employed a systematic grid‐based sampling design, dividing the region into 1 km × 1 km unit grids. This design ensures spatial sampling uniformity, following representative camera trap sampling principles. Notably, camera traps were deployed based on grid centroids without targeting linear features such as trails or roads, ensuring a non‐trail‐dependent sampling framework that avoids detection biases associated with path‐preferring species (Greco et al. 2025). Within each grid, 1–2 infrared camera traps (E3H series, Shenzhen Ereagle Technology Co. Ltd., Shenzhen, China; Ltl 6511 series, Zhuhai Ltl Acorn Electronics Co. Ltd., Zhuhai, China) were deployed. To ensure spatial independence, the distance between any two cameras was maintained at over 500 m. A total of 122 infrared camera traps were installed, comprehensively covering major vegetation types within the study area (including tropical evergreen monsoon forest, tropical montane rainforest, tropical semi‐deciduous monsoon forest, and tropical valley rainforest), thereby enabling effective collection of activity rhythm data for wildlife such as the Asiatic brush‐tailed porcupine across different habitats (Figure 1). Each infrared camera trap operated continuously with 24‐h functionality. Devices were configured with a medium sensitivity setting and programmed to capture 3 consecutive still images plus a 10‐s video sequence upon each triggering event. The burst mode featured a 10‐s interval between consecutive captures, with a 30‐s minimum trigger reactivation period. These cameras were checked for working conditions every 3 months, from October 2020 to October 2022 (2 years). Camera installation was conducted from August to September 2020, taking approximately 2 months. Given the mountainous terrain and dense vegetation, the work was performed by a cross‐institutional team: researchers from universities and institutes handled technical tasks (grid division, position calibration), while staff from Jianfengling Branch Bureau of Hainan Tropical Rainforest National Park provided terrain guidance and field support. This collaboration ensured scientific sampling, improved efficiency, and mitigated field risks. During the monitoring period, quarterly maintenance was conducted by the same team: replacing batteries, exporting data, securing devices, cleaning lenses, and on‐site debugging to maintain consistent sensitivity and shooting modes, preventing data loss. To contextualize interspecific interactions, we estimated densities of the target species, competitors, predators, and domestic dogs (anthropogenic disturbance proxy) via a grid‐based density estimation method validated for unmarked tropical mammals (Wang et al. 2019). This method was applied based on the aforementioned systematic grid‐based sampling design, using the grid units defined in the sampling framework to calculate species density per km^2^. Specifically, this approach calculates density (individuals/km^2^) using the maximum number of independently identified individuals per 1 km × 1 km grid—a practical alternative to mark‐recapture (logistically unfeasible here due to dense vegetation). The estimated densities of focal species are as follows: Asiatic brush‐tailed porcupine (0.32 ± 0.08 individuals/km^2^, identified by quill patterns and body size), ferret‐badger (competitor, 0.21 ± 0.05 individuals/km^2^), leopard cat (predator, 0.04 ± 0.02 individuals/km^2^), and domestic dog (anthropogenic disturbance proxy, 0.15 ± 0.04 individuals/km^2^).

To process the large dataset and mitigate potential pseudoreplication, a standardized four‐step workflow was adopted:

1. Preliminary screening: All media files were imported into Excel databases linked to camera IDs, timestamps; ~7% of total data (invalid captures, e.g., wind/vegetation‐triggered empty images, overexposed/underexposed files) were manually removed.
2. Species identification: Clear captures were distinguished based on species‐specific morphological traits (e.g., distinct quill patterns for Asiatic brush‐tailed porcupine, unique facial markings for ferret‐badger); for unclear captures, a team conducted cross‐verification, and unidentifiable records were discarded.
3. Target filtering: Only records of the four focal species (Asiatic brush‐tailed porcupine, ferret‐badger, leopard cat, domestic dog) were retained, with redundant data discarded to match study objectives.
4. Pseudoreplication mitigation: All consecutive triggers recorded at the same camera station within a 30‐min interval were consolidated into a single independent valid event (O'Brien et al. 2003).

### 2.3 Data Analysis

To ensure clarity and consistency, three core metrics were defined for this study (Table 1), with no redundant or overlapping indices. All subsequent analyses strictly adhere to these definitions.

**TABLE 1:** Definitions, calculations, and purposes of core metrics.

Metric name	Definition	Calculation	Purpose
Relative activity index	Quantifies the activity intensity of the Asiatic brush‐tailed porcupine across different time windows	RAI = (Ti/Ni) × 100% Ti = Number of independent detections in a specific time window (e.g., full moon, December). Ni = Total independent detections of the porcupine during the entire study period	Analyze the effects of lunar phases, seasons, and temperature on the porcupine's activity intensity
Relative detection frequency	Quantifies the relative activity level of sympatric species, serving as a proxy for interspecific pressure	Relative Detection Frequency = (Number of independent detections of the sympatric species/Total valid camera‐days) × 1000	Evaluate the intensity of interspecific competition/predation pressure; used as a continuous covariate to quantify sympatric species' presence in GLMs (following Iannarilli et al. 2025)
Grid‐based density estimation method	Quantifies the population density of focal species, reflecting their spatial distribution concentration	Relative Detection Frequency = Maximum number of independently identified individuals per 1 km × 1 km grid/Grid area (km^2^)	Provide ecological context for interspecific interactions (e.g., higher density of ferret‐badgers indicates stronger competitive pressure)

All statistical analyses and data processing were conducted in *R* version 4.4.1 (R Core Team 2021). Specific statistical tools and their corresponding R packages are cited throughout the section, ensuring methodological transparency and reproducibility. The analytical workflow was structured to address the study's core objectives, activity rhythm quantification, temporal overlap assessment, environmental correlation analysis, and interspecific interaction modeling, as detailed below:

#### 2.3.1 Data Preprocessing

1. Gregorian dates from camera metadata were converted to lunar calendar dates, with the lunar cycle divided into five phases (new moon: lunar days 1–8; waxing crescent: 9–11; full moon: 12–19; waning gibbous: 20–23; waning crescent: 24–30), (Sutherland and Predavec 1999). These phases were further grouped into three illumination levels: low (new moon + waning crescent), medium (waxing crescent + waning gibbous), and high (full moon).
2. The study period was split into dry (October–April) and wet (May–September) seasons based on local climatic characteristics.
3. Temperature data were extracted directly from camera photo metadata to ensure real‐time matching with species detection events.
4. The presence of sympatric species (leopard cat, ferret‐badger, and domestic dog) was quantified using “relative detection frequency”, calculated as (number of independent detections of the species/total valid camera‐days) × 1000. This continuous covariate reflects the relative activity level of each species in the study area (rather than a binary detection/non‐detection variable), with leopard cat frequency indicating relative predation pressure, ferret‐badger frequency indicating relative competitive intensity, and domestic dog frequency indicating relative anthropogenic disturbance intensity, consistent with the covariate integration approach recommended by Iannarilli et al. (2025).
5. Gregorian dates were converted to lunar calendar dates, with full moon explicitly defined as lunar days 12–19 (Sutherland and Predavec 1999). This classification directly aligns with our research question on full‐moon‐induced activity suppression and crepuscular compensation. We separately analyzed activity patterns (via KDE) and intensity (via RAI) for full‐moon periods to test the hypothesis.

#### 2.3.2 Quantification of Diel Activity Rhythms

KDE was applied to quantify the diel activity patterns of the four focal species using the activity package (Rowcliffe et al. 2014):

KDE was used to generate standardized daily activity probability density curves, describing the temporal distribution of the species' activity (e.g., crepuscular vs. late‐night concentration); (Ridout and Linkie 2009). Notably, KDE was not used to compare activity intensity (consistent with Iannarilli et al. 2025); instead, activity intensity was quantified via the Relative Activity Index (RAI) (Section 2.3.4), following the combined framework of Rowcliffe et al. (2014) and Chen et al. (2019).

Activity rhythms were compared across two temporal dimensions (lunar phase cycles and seasonal cycles) to identify plasticity in response to environmental variation.

#### 2.3.3 Temporal Overlap and Statistical Comparison

The coefficient of overlap (Δ) proposed by (Ridout and Linkie 2009) was calculated to evaluate similarity in activity patterns using the overlap package (Meredith and Ridout 2021):

Δ ranges from 0 (no temporal overlap) to 1 (complete synchrony), representing the cumulative overlapping area under KDE curves across a 24‐h cycle.

Statistical differences in activity rhythms were assessed using the compareCkern() function from the activity package (Rowcliffe et al. 2014):

A Wald test framework supplemented with 1000 bootstrap iterations was used to determine the significance (*α* = 0.05) of rhythm variations among species, lunar phases, and seasons.

#### 2.3.4 Relative Activity Index (RAI) and Temperature Correlation

Following the framework of Chen et al. (2019), the RAI was used to quantify Asiatic brush‐tailed porcupine activity intensity across lunar phases and months:

Formula: RAI = (Ti/Ni) × 100%, where Ti = number of valid detections in a given timestamp (lunar phase/month), and Ni = total valid detections of the species across the entire study period.

Statistical tests were performed using base R functions (R Core Team 2021):

Kruskal–Wallis test to assess overall differences in RAI across lunar phases; post hoc Mann–Whitney U tests were applied for pairwise comparisons if significant differences were detected (α = 0.05).

Normality test was performed on monthly RAI values to verify their compliance with the normality assumption of parametric statistical tests. Since the test confirmed normality (*p* > 0.05), Pearson correlation analysis was used to examine the relationship between monthly average temperature and monthly RAI—this analysis not only quantifies the thermal constraint on porcupine activity, but also provides insights for addressing our third research question. Specifically, by linking RAI reductions and activity window compression under extreme temperatures to potential physiological costs (e.g., energetic stress, water loss) and adaptive limitations, we infer how behavioral thermoregulation trades off with long‐term survival under environmental change. This normality requirement applies to parametric correlation analysis, not to the subsequent Generalized Linear Models (GLMs), which do not require residual normality.

#### 2.3.5 Modeling Interspecific Interactions Across Lunar Illumination Levels

GLMs were constructed to investigate how sympatric species influence the Asiatic brush‐tailed porcupine's activity intensity under different moonlight conditions, with model optimization conducted using base R functions (R Core Team 2021):

Z‐score standardization of continuous predictors to mitigate scale‐related biases.

Variance Inflation Factor (VIF) analysis to address multicollinearity; variables with VIF > 5 were excluded (Fox and Weisberg 2019).

Fligner–Killeen test to detect and remove outliers, enhancing model reliability.

Due to significant overdispersion and violation of Poisson distribution assumptions, a negative binomial regression model was adopted using the MASS package (Venables and Ripley 2002) to better fit the data characteristics. Model outputs included estimates, standard errors, *z*‐values, and *p*‐values to quantify the strength and significance of interspecific effects.

## 3 Results

During the study period, infrared camera traps accumulated a total of 169,812 camera‐days of monitoring effort. Within this period, a total of 4508 independent detections of the brush‐tailed porcupine were recorded. Other species detected included the leopard cat with 109 independent detections (relative detection frequency = 0.64 ± 0.08 detections/1000 camera‐days), the domestic dog with 191 independent detections (relative detection frequency = 1.12 ± 0.15 detections/1000 camera‐days), and the ferret‐badger with 1164 independent detections (relative detection frequency = 6.85 ± 0.32 detections/1000 camera‐days).

### 3.1 Annual Activity Rhythms

**FIGURE 2:**
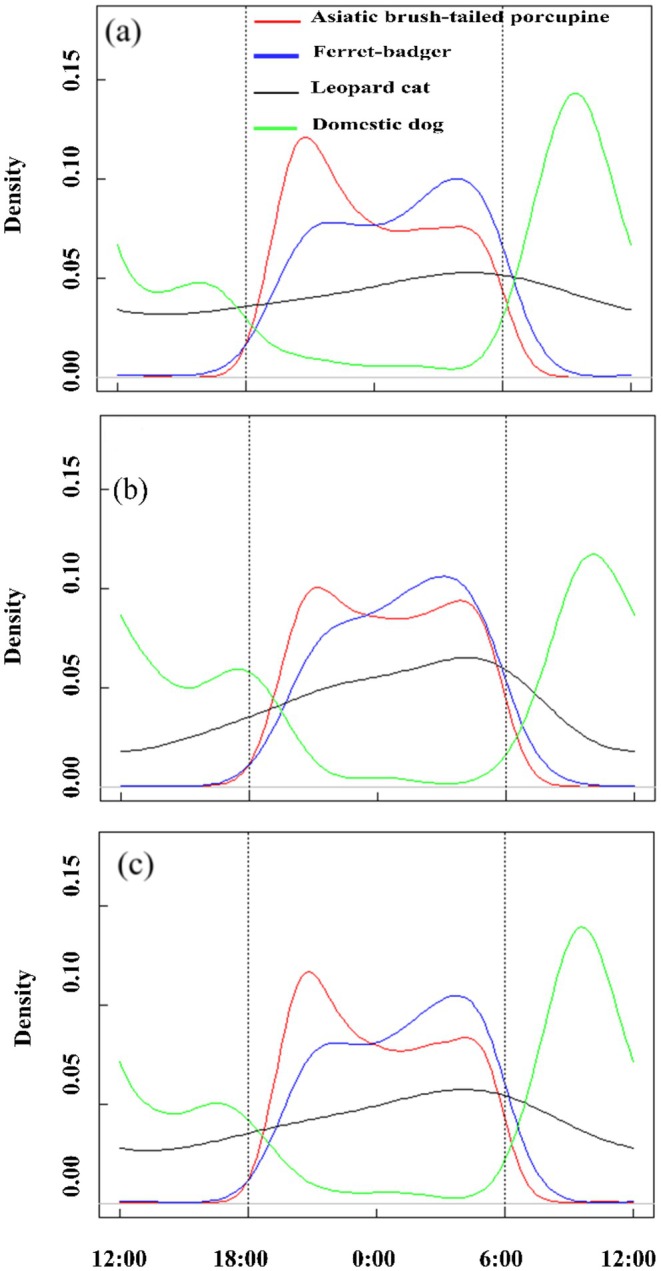
Daily activity rhythms of the brush‐tailed porcupine, leopard cat, domestic dog, and the ferret‐badger during the dry season (a), the rainy season (b), and throughout the year (c) in the Jianfengling area of Hainan Tropical Rainforest National Park from 2020 to 2022.

Over the annual cycle, the brush‐tailed porcupine consistently demonstrated typical nocturnal behavior with a unimodal activity pattern (Figure 2c). The leopard cat, being nocturnal, peaked between 04:00 and 06:00, resulting in a moderate overlap coefficient of Δ = 0.632 with the porcupine and a significant difference in their activity cycles (*p* < 0.01). The ferret‐badger, also nocturnal, reached its activity peak between 03:00 and 05:00, showing a significant difference in peak timing from the porcupine (*p* < 0.01). Conversely, the domestic dog was distinctly diurnal, with a low overlap coefficient (Δ < 0.5; Figure 2c). Dietary differentiation supports this coexistence: ferret‐badgers rely primarily on insects and small vertebrates (Zheng and Song 2010), while porcupines are strictly vegetarian (Wang et al. 2019), eliminating direct food competition. Notably, low interspecific food competition supports such activity overlap: porcupines adjust their diet seasonally (fruits/new leaves in wet season, bark/tubers in dry season) with overlapping core food sources (Wang et al. 2019), so seasonal activity shifts are primarily driven by temperature/humidity rather than food limitation. This annual baseline facilitates the interpretation of seasonal activity shifts in the following section.

### 3.2 Seasonal Variation in Activity Rhythms

The Asiatic brush‐tailed porcupine exhibited distinct seasonal plasticity in diel activity patterns, while sympatric species (leopard cat, ferret‐badger, domestic dog) maintained relatively stable nocturnal traits (Figure 2).

During the dry season, the brush‐tailed porcupine displayed a unimodal nocturnal activity pattern: activity began between 18:00 and 19:00, peaked from 20:00 to 22:00, remained consistent from 23:00 to 06:00, and was absent from 07:00 to 17:00 (Figure 2a). The leopard cat exhibited typical nocturnal behavior, with activity peaking between 04:00 and 06:00. The overlap coefficient with the porcupine was Δ = 0.654, and their activity rhythms differed significantly (*p* < 0.01; Figure 2a). Similarly, the ferret‐badger was nocturnal, peaking between 03:00 and 05:00, and showed a significant difference in activity patterns compared with the porcupine (*p* < 0.01; Figure 2a). In contrast, the domestic dog had low overlap with the porcupine (Δ < 0.5; Figure 2a).

In the rainy season, the brush‐tailed porcupine remained nocturnal but adopted a bimodal activity pattern, with peaks around 04:00 and 21:00 (Figure 2b), and showed a significant change from dry season patterns (*p* < 0.01). The leopard cat maintained similar nocturnal characteristics as in the dry season, with no significant change (*p* = 0.636). It peaked between 04:00 and 06:00, had an overlap of Δ = 0.702 with the porcupine, and displayed a significant difference in activity (*p* < 0.01; Figure 2b). The ferret‐badger continued to be nocturnal, peaking between 03:00 and 05:00, and exhibited significant variation from the porcupine's temporal patterns (*p* < 0.01; Figure 2b). The domestic dog again showed minimal overlap with the porcupine (Δ < 0.5; Figure 2b).

### 3.3 Lunar Phase Variation in Activity Rhythms

**FIGURE 3:**
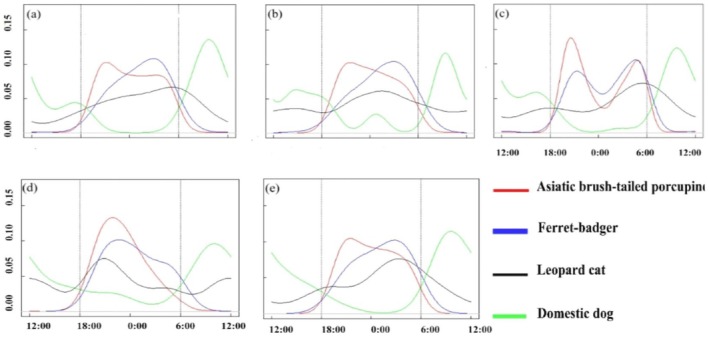
Daily activity rhythms of the brush‐tailed porcupine, leopard cat, domestic dog, and the ferret‐badger during the different Lunar Phase Cycles in the Jianfengling area of Hainan Tropical Rainforest National Park from 2020 to 2022. (a) new moon, (b) waxing moon, (c) full moon, (d) waning gibbous, and (e) waning crescent.

The porcupine's activity rhythm and intensity showed marked responses to lunar cycles, while sympatric species exhibited variable adjustments to moonlight intensity (Figure 3).

During the new moon phase, the brush‐tailed porcupine and ferret‐badger showed temporally separated activity peaks: the porcupine peaked between 20:00 and 22:00, while the ferret‐badger peaked from 03:00 to 04:00. The leopard cat was most active in the early morning, and the domestic dog remained largely inactive at night (Figure 3a).

In the waxing moon phase, both the brush‐tailed porcupine and ferret‐badger reached their activity peaks during the latter half of the night, specifically between 03:00 and 04:00. In contrast to other lunar phases, the leopard cat and domestic dog showed increased nocturnal activity (Figure 3b).

Under the full moon, both the brush‐tailed porcupine and ferret‐badger exhibited bimodal activity patterns. The porcupine peaked at 20:00–21:00 and 04:00–05:00, while the ferret‐badger peaked at 21:30–22:00 and 03:30–04:30. The leopard cat maintained its early morning peak, and the domestic dog showed minimal nocturnal activity (Figure 3c). Notably, the 04:00–05:00 peak represents brief crepuscular supplementary activity, which might be a behavioral adjustment to avoid high predation risk from visually hunting predators (e.g., leopard cat) during full‐moon nights. In other lunar phases (e.g., new moon, waning crescent) the porcupine concentrated activity in nocturnal periods (20:00–22:00) with no obvious crepuscular activity (Figure 3a,e).

During the waning gibbous phase, the brush‐tailed porcupine peaked between 22:00 and 23:00, the ferret‐badger from 23:00 to 00:00, and the leopard cat from 21:00 to 22:00. The domestic dog was relatively active in the early part of the night (Figure 3d).

In the waning crescent phase, the brush‐tailed porcupine peaked from 20:00 to 22:00, the ferret‐badger from 03:00 to 05:00, and the leopard cat from 03:00 to 04:00. The domestic dog continued to show little nocturnal activity (Figure 3e).

### 3.4 Relative Activity Index

**FIGURE 4:**
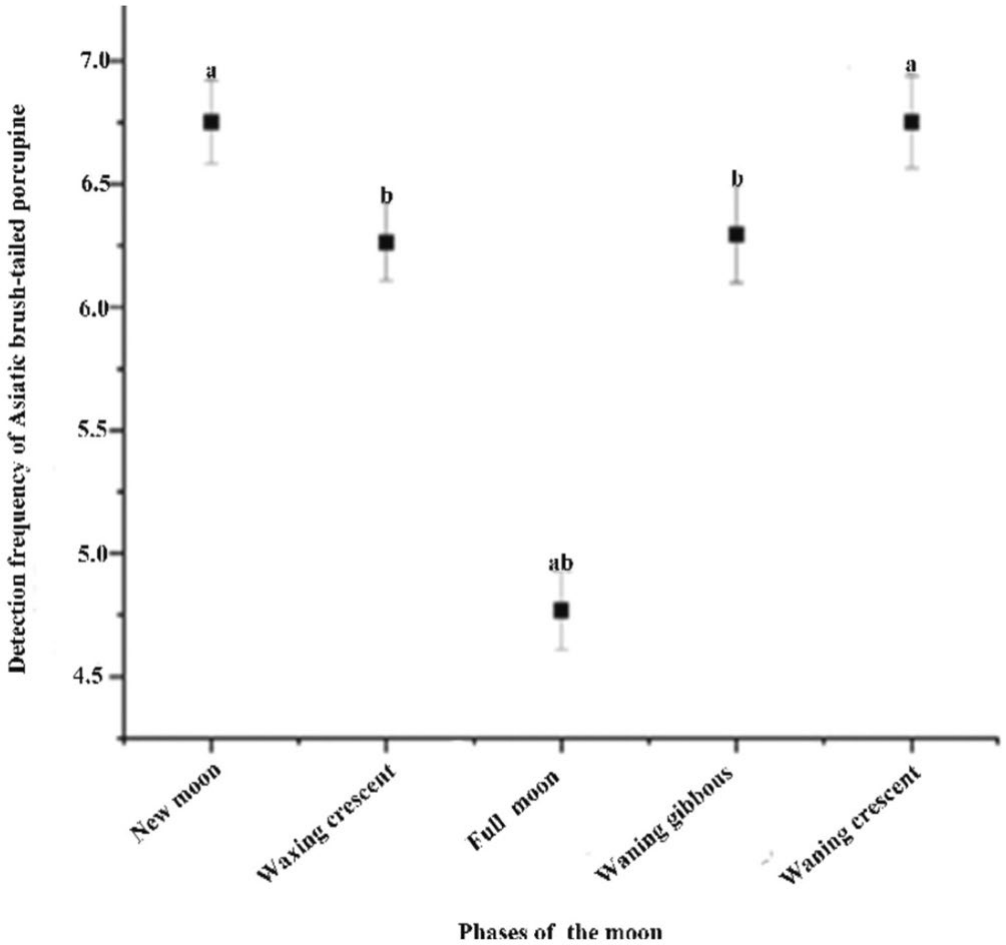
The Relative Activity Index of brush‐tailed porcupines varied across lunar phases in the Jianfengling area of Hainan Tropical Rainforest National Park from 2020 to 2022. Different letters indicated statistically significant differences (*p* < 0.05; Mann–Whitney *U* test).

The daily RAI of brush‐tailed porcupines varied across lunar phases, averaging (6.75 ± 0.17) daily RAI during the new moon, (6.26 ± 0.15) in the waxing moon, (4.77 ± 0.16) under the full moon, (6.30 ± 0.20) in the waning gibbous, and (6.75 ± 0.19) in the waning crescent phase. A Kruskal–Wallis test revealed significant differences in RAI among these lunar phases (χ^2^ = 18.2, df = 4, *p* < 0.01). Post hoc pairwise comparisons (Mann–Whitney U test) indicated statistically significant differences between the following phase pairs: waning crescent and waxing moon, waning crescent and waning gibbous, new moon and waxing moon, and new moon and waning gibbous (Figure 4).

**FIGURE 5:**
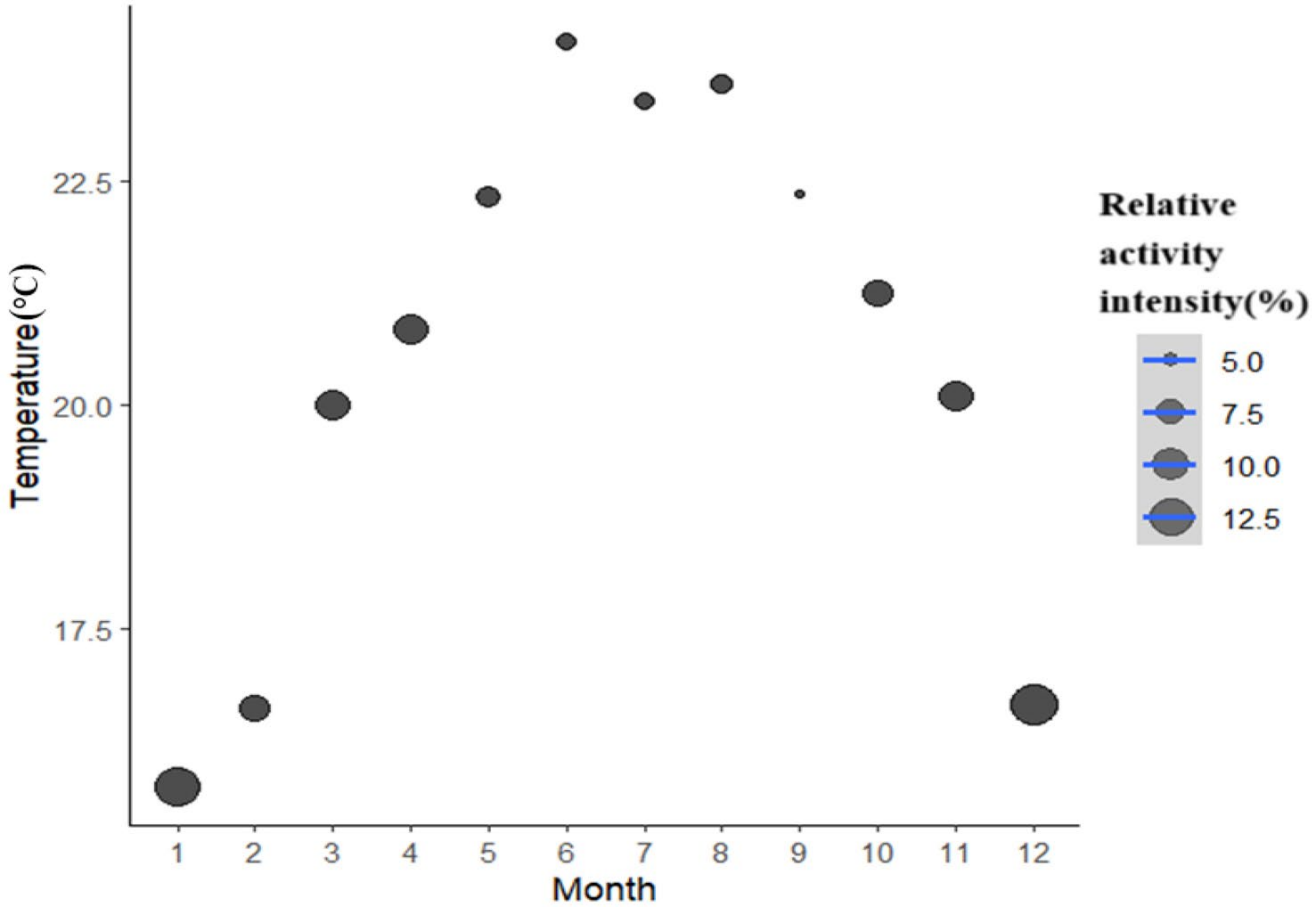
Correlation between the Relative Activity Index of the brush‐tailed porcupines in relation to temperature in the Jianfengling area of Hainan Tropical Rainforest National Park from 2020 to 2022.

**FIGURE 6:**
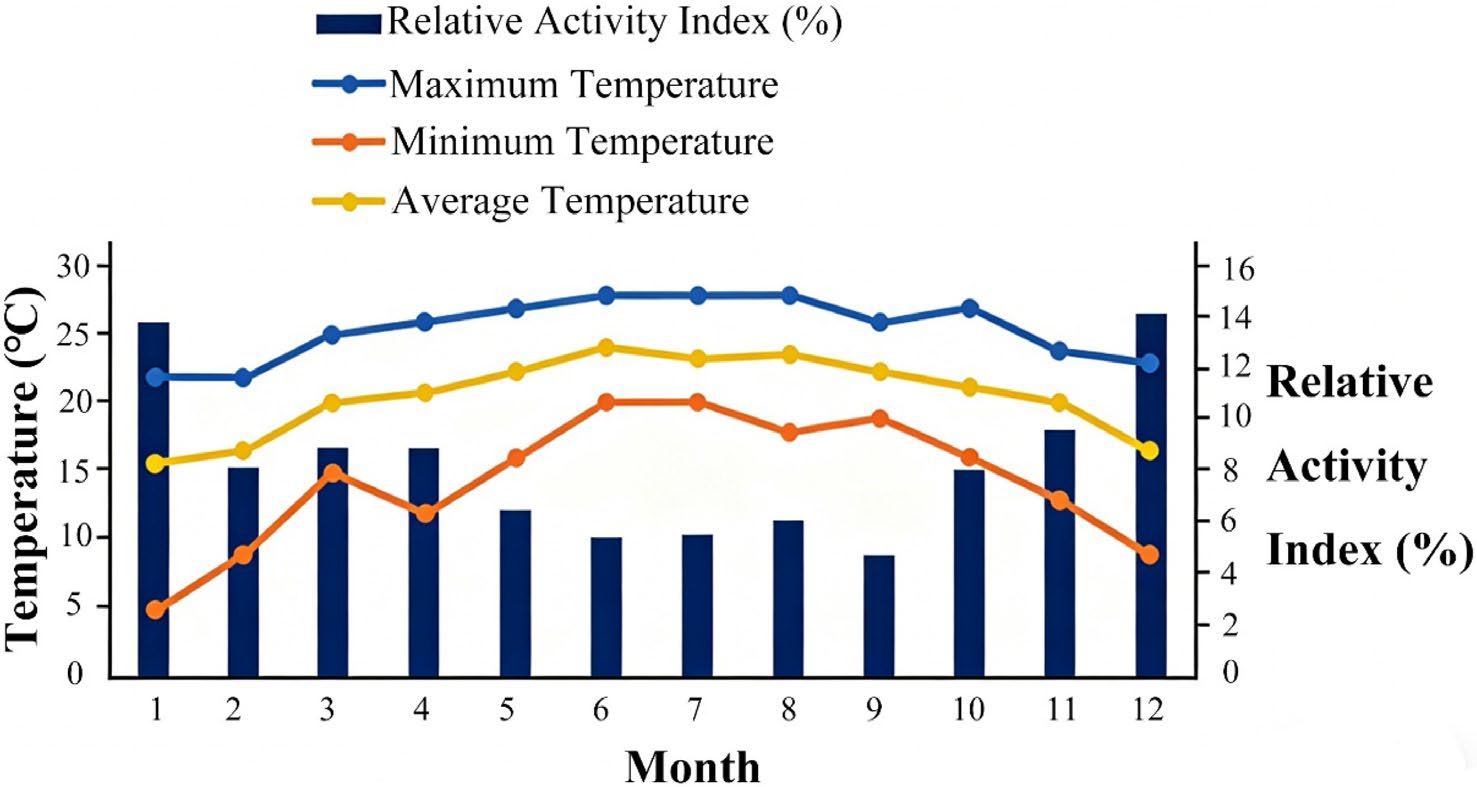
Monthly temperature distribution (maximum, minimum, and average temperature) and the optimal temperature range associated with peak activity of the Asiatic brush‐tailed porcupine in the Jianfengling area (2020–2022).

As shown in Figure 5, the brush‐tailed porcupine reached its peak activity in December and its lowest level in September. Normality tests confirmed that the monthly RAI values followed a normal distribution. This result justified the use of Pearson correlation analysis for examining temperature‐RAI relationships (Figure 5). For the GLM analysis of interspecific interactions, we used negative binomial regression (GLM) to address overdispersion, as Poisson regression was inappropriate due to violated distribution assumptions. A significant negative correlation was observed between temperature and monthly RAI (*t* = −3.827, *p* < 0.001). The optimal temperature range for porcupine activity was 15°C–22°C, with most activity occurring below the monthly average temperature (Figure 6).

GLMs indicated that the relationship between the porcupine's activity intensity and interspecific interactions varied with lunar illumination intensity. For the initial model construction, we incorporated the study‐area average relative detection frequency of sympatric species as continuous predictors (standardized via z‐score to mitigate scale biases), with these single constant values per species quantified as: leopard cat (0.64 ± 0.08 detections/1000 camera‐days, proxy for overall predation pressure), ferret‐badger (6.85 ± 0.32 detections/1000 camera‐days, proxy for overall competitive intensity), and domestic dog (1.12 ± 0.15 detections/1000 camera‐days, proxy for overall anthropogenic disturbance). These average values reflect the general intensity of interspecific pressures across the entire study area and monitoring period, and the model stratification by lunar illumination levels (low/medium/high) further accounted for temporal variation in pressure associated with moonlight‐driven behavioral shifts of sympatric species. Ferret‐badgers exhibited the highest average relative activity across the study area, indicating the strongest overall competitive pressure, while leopard cats had the lowest average activity, suggesting moderate overall predation pressure. We acknowledge that this single average value approach per species may underestimate fine‐scale spatial and temporal (seasonal/site‐specific) variation in the activity of potential competitors and predators, a key limitation of the current analysis. As suggested, incorporating the site‐varying and seasonal RAI of sympatric species as dynamic predictors in future GLM analyses would better account for spatial heterogeneity in habitat use and temporal shifts in activity of leopard cats, ferret‐badgers, and domestic dogs, thereby refining the quantification of interspecific pressure variation and its effects on porcupine activity. During the low illumination period, the porcupine significantly avoided predators (*z* = −3.022, *p* < 0.01) and domestic dogs (*z* = −2.320, *p* < 0.05), but showed a positive relationship with competitor occurrence (*z* = 3.090, *p* < 0.01). This positive association may be attributed to the high relative activity of ferret‐badgers, leading to unavoidable temporal overlap when resources are concentrated, even as the porcupine avoids predators. Under both high and medium illumination levels, activity intensity was positively associated with competitor presence (high: *z* = 4.891, *p* < 0.01; medium: *z* = 4.891, *p* < 0.01). (Table 2).

**TABLE 2:** Generalized linear model analysis of Relative Activity Index—The Effects of leopard cat, domestic dog, and the ferret‐badger on the Activity Intensity of the brush‐tailed porcupines during the phase of the moon in the Jianfengling area of Hainan Tropical Rainforest National Park from 2020 to 2022.

Period/variable	Estimate	Std.Error	*z*‐value	Pr (>|z|)
Low illumination period				
Intercept	5.729	0.499	11.492	< 0.01
Leopard cat	−0.232	0.077	−3.022	< 0.01
Ferret‐badger	0.015	0.005	3.090	< 0.01
Domestic dog	−0.121	0.060	−2.020	< 0.05
Medium illumination period				
Intercept	2.861	0.398	7.189	< 0.01
Leopard cat	0.141	0.121	1.166	0.244
Ferret‐badger	0.057	0.014	4.096	< 0.01
Domestic dog	0.046	0.174	0.265	0.791
High illumination period				
Intercept	3.113	0.294	10.590	< 0.01
Leopard cat	−0.051	0.089	−0.572	0.567
Ferret‐badger	0.055	0.011	4.891	< 0.01
Domestic dog	0.088	0.260	0.337	0.736

## 4 Discussion

This study used infrared camera traps technology to investigate trade‐off strategies in the activity rhythms of the Asiatic brush‐tailed porcupine in tropical rainforest habitats. The results revealed clear seasonal variation in its daily activity pattern, which was predominantly nocturnal with brief crepuscular supplementary activity only under high predation risk (e.g., full‐moon phase). This adjustment likely balances foraging needs and predation avoidance, consistent with the expectation that nocturnal activity is prioritized during low moonlight periods to reduce detectability. When facing predators, competitors, and human disturbance, the brush‐tailed porcupine adopted a safety‐priority strategy, shifting its active periods to avoid risks, albeit at the cost of increased foraging time and difficulty, inferred from the compressed nocturnal activity window and reduced overlap with optimal foraging periods (Section 3.2, 3.3).

The brush‐tailed porcupine exhibited a unimodal activity pattern during the dry season, peaking around 20:00, and shifted to a bimodal pattern in the rainy season, with peaks at approximately 21:00 and 04:00. In the dry season, resource scarcity and compressed activity windows likely forced the porcupine to rely more on less efficient foraging modes, such as olfactory and auditory cues, resulting in higher activity intensities at peak times compared with the wet season. These findings are consistent with the “risk–energy trade‐off” hypothesis (Ridout and Linkie 2009): We infer that the porcupine may conserve energy by concentrating activity in the dry season, while increased predation pressure in the rainy season may have led to a bimodal strategy to balance resource use and risk avoidance.

From an ecological and evolutionary adaptation perspective, the lunar cycle operated as a key selective force shaping the activity rhythm of the brush‐tailed porcupine. This species has evolved a distinct lunar avoidance strategy, whereby its activity frequency declines significantly with increasing lunar illumination. The lowest activity occurs during the full‐moon phase (4.77 ± 0.16 events per day), while peak activity is observed during the new and waning moon phases (6.75 ± 0.17 events per day). This behavioral pattern may represent a potential adaptive response to predation pressure, facilitated by a sensory system dominated by olfactory and auditory cues—traits that reduce detectability to visually hunting predators such as the leopard cat (Prugh and Golden 2014). Temporal niche partitioning is evident: the leopard cat is most active between 04:00 and 06:00, whereas the porcupine concentrates its activity in the early night (20:00–22:00), thereby minimizing temporal overlap with this predator. However, GLM results revealed a seemingly counterintuitive pattern: leopard cats significantly suppressed porcupine activity during low lunar illumination (*z* = −3.022, *p* < 0.01) but had no detectable suppressive effect during the full moon (high illumination, *z* = −0.051, *p* = 0.567). This pattern is not a contradiction but rather a product of two complementary ecological mechanisms, combined with the methodological limitation of our GLM variable design (single study‐area average values for sympatric species activity, which oversimplifies fine‐scale spatial and temporal variability in leopard cat and porcupine activity). First, the porcupine's lunar avoidance strategy reduces its overall activity intensity during the full moon (the lowest RAI across all lunar phases), which likely minimizes direct encounters with leopard cats to avoid predation risk altogether—rather than exhibiting targeted avoidance of the predator's activity peak. Second, the porcupine exhibits a crepuscular compensation strategy during the full moon: it shifts its remaining activity to dawn/dusk periods (04:00–05:00), which only partially overlaps with the leopard cat's early morning activity peak (04:00–06:00), thus reducing the predator's behavioral impact on porcupine activity intensity. Collectively, these two strategies eliminate the detectable suppressive effect of leopard cats in the full‐moon phase, while the porcupine's higher overall activity during low illumination increases its exposure to leopard cat predation risk, resulting in the significant suppression observed in the model. Notably, this apparent differential response may also be amplified by our use of single average relative detection frequency values for leopard cats (excluding fine‐scale spatial/temporal variation in predator activity across lunar phases and sites); a more nuanced model incorporating site/seasonally varying RAI for sympatric species (as suggested) would likely refine our understanding of the porcupine's predator avoidance responses across lunar illumination levels. GLMs thus confirmed a context‐dependent suppressive effect of leopard cat presence on porcupine activity, modulated by lunar illumination and the porcupine's adaptive behavioral strategies. This form of lunar avoidance is widely documented among non‐visual mammals (Prugh and Golden 2014) and reflects an evolved trade‐off: although it may reduce short‐term foraging efficiency, it confers long‐term fitness benefits by lowering predation risk. Similar adaptive behaviors had been reported in other small prey, including Stephens' kangaroo rat ( *Dipodomys stephensi* ) (Shier et al. 2020), with lunar‐driven behavioral adjustments also documented in mesocarnivores such as the Eurasian otter ( *Lutra lutra* ) (Shi et al. 2021).

The brush‐tailed porcupine exhibited a strong inverse relationship between its activity intensity and ambient temperature, with an optimal thermal range of 15°C–22°C. This range fell substantially below the average nighttime temperatures of its tropical rainforest habitat. This finding directly addresses the third research question regarding physiological costs and adaptive limitations of behavioral thermoregulatory strategies. This result indicates that the porcupine's activity rhythms are tightly constrained by thermal conditions, and prolonged nocturnal warming (a key consequence of climate change) may exceed its behavioral adaptive capacity—imposing substantial physiological costs such as increased water loss and energetic stress (McNab 1980), especially during dry periods when the porcupine is speculated to rely on preformed water in its diet as its main source of hydration—a pattern we inferred from activity reductions in high‐temperature months, rather than direct measurements of water metabolism or energetic expenditure. This could indirectly limit its foraging efficiency, but the specific physiological costs remain unconfirmed in this study. The absence of drinking behavior in field observations further indicated that curtailing activity during thermally stressful periods would be a key water conservation strategy. In the context of climate change, this thermoregulatory strategy faces serious threats; increasing nocturnal temperatures, prolonged droughts, and diminished food resources might intensify niche overlap with other species, potentially reducing the porcupine's ecological resilience and posing indirect risks to long‐term population persistence (inferred from its constrained activity thermal range and behavioral plasticity limits), ultimately jeopardizing the porcupine's ecological resilience and population viability.

The peak activity periods of the brush‐tailed porcupine and its primary competitor, the ferret‐badger, showed a clear temporal divergence. The ferret‐badger was most active from 03:00 to 05:00, whereas the brush‐tailed porcupine primarily engaged in activity from 20:00 to 22:00. This chronological segregation may indirectly reduce direct resource competition, as it minimizes temporal overlap during peak foraging periods (inferred from the species' dietary overlap and activity peaks). During the waning lunar phase, when predation risk was lower, their activities were positively correlated, potentially indicating behavioral flexibility. In periods hypothesized to have abundant resources, they may tolerate limited competition to maximize foraging efficiency. Conversely, under resource‐scarce conditions during the full moon, they were compelled to confront the ferret‐badger directly, adopting a “survival at the cost of energy” trade‐off strategy that highlighted their behavioral plasticity in response to competitive stress.

As extensions of anthropogenic disturbance, domestic dogs notably suppressed the activity intensity of brush‐tailed porcupines, with the strongest effect observed during the low illumination period. The dogs' vocalizations, footfalls, and olfactory cues may potentially disrupt the porcupines' sensory environment, compromising their ability to forage and evade predators (Hughes and Macdonald 2013). In response, the porcupines adopted a temporal compression strategy, shifting their activity to late‐night hours to avoid exposure. Although this “temporal avoidance” was an effective short‐term coping mechanism, it may potentially carry long‐term ecological consequences, including diminished foraging success, reduced reproductive opportunities, and a narrower chronological niche (Hughes and Macdonald 2013). Notably, we cannot definitively confirm these long‐term consequences in the context of our study, as our data are limited to activity pattern observations rather than direct measurements of key parameters such as foraging time, foraging efficiency, and reproductive output. These potential outcomes remain a plausible inference based on ecological theory and indirect evidence, but their validation requires supplementary research. Future studies should incorporate quantitative assessments of foraging duration and success to verify whether the observed temporal shift indeed translates to the hypothesized long‐term effects, thereby strengthening the link between anthropogenic disturbance, behavioral adaptation, and ecological consequences.

Against the escalating pressures of global climate warming and anthropogenic disturbance, elucidating the adaptive mechanisms governing the activity rhythms of Asiatic brush‐tailed porcupines provides targeted empirical support for its conservation: (1) given its optimal activity temperature (15°C–22°C) prioritize preserving montane rainforest habitats with stable microclimates (e.g., retaining canopy cover to mitigate nocturnal warming); (2) its strong avoidance of full‐moon phases and sensitivity to free‐ranging domestic dogs suggest restricting non‐essential human activities (e.g., routine patrols, eco‐tourism) in core habitats during full moons and controlling free‐ranging dog access (e.g., wildlife‐friendly fencing) to reduce sensory disruption; (3) its temporal niche segregation with ferret‐badgers highlights the need to maintain habitat connectivity for both species, avoiding fragmentation‐driven competitive stress. These targeted measures directly inform adaptive management of the species in tropical rainforest ecosystems.

In summary, this study focused on the brush‐tailed porcupine in Hainan Island's Jianfengling National Tropical Rainforest Park. Using infrared camera data, we systematically analyzed its activity rhythms to infer the complex regulatory mechanisms and adaptive strategies shaped by multiple ecological pressures. As a typical nocturnal species, the porcupine has reduced visual acuity compensated by enhanced olfactory and auditory abilities. By prioritizing survival over short‐term energy gain, the species avoids bright moonlight, extreme temperatures, and direct competition, maintaining a balance amid various ecological stresses. However, the combined effects of climate change and human activities may exceed the limits of its behavioral adaptability, threatening long‐term population survival. This research not only provides insights into the mechanisms of behavioral adaptations in tropical rainforest ecosystems but also offers a scientific basis for developing comprehensive conservation strategies for endangered species amid global environmental change. Notably, all mechanisms proposed in this discussion are inferred from observed activity patterns, as we did not directly measure key parameters such as predation events, interspecific competition intensity, energy expenditure, or survival rates. Thus, the causal links between environmental factors (e.g., lunar illumination, temperature) and porcupine activity strategies remain speculative. For example, while lunar avoidance is consistent with predation risk avoidance, we cannot rule out other unmeasured factors (e.g., prey availability for leopard cats) that may co‐vary with moonlight. Future studies integrating direct measurements of predation, resource use, and physiological costs will help verify these inferred mechanisms.

## Author Contributions

**Haidong Zhou:** writing – original draft (lead). **Wenbo Yan:** funding acquisition (lead), writing – review and editing (lead). **Zhigao Zeng:** funding acquisition (lead), writing – review and editing (lead). **Shaoliang Xue:** investigation (equal). **Qi Wang:** formal analysis (equal). **Chunshen Liang:** investigation (equal). **Lu Liang:** investigation (equal).

## Funding

This research was funded by the Hainan Institute of National Park, grant numbers KY‐21ZK01 and KY‐24ZK01, and the Jianfengling Branch of the Management Office of Hainan Tropical Rainforest National Park, grant number: Donghe (Ke) 2020–0312.

## Ethics Statement

Ethical review and approval were waived for this study because infrared camera traps facilitate valuable data without disturbing wildlife or compromising ethical considerations.

## Consent

The authors have nothing to report.

## Conflicts of Interest

The authors declare no conflicts of interest.

## Data Availability

I confirm that the Data Availability Statement is included in the main file of my submission, and that access to all necessary data files is provided to editors and reviewers. The data are requested for the peer review process. https://doi.org/10.6084/m9.figshare.30343792.
